# Alterations in Pharmacokinetics of Gemcitabine and Erlotinib by Concurrent Administration of Hyangsayukgunja-Tang, a Gastroprotective Herbal Medicine

**DOI:** 10.3390/molecules22091515

**Published:** 2017-09-10

**Authors:** Tae Hwan Kim, Soyoung Shin, Sarah Kim, Jürgen B. Bulitta, Kwon-Yeon Weon, Sang Hoon Joo, Eunsook Ma, Sun Dong Yoo, Gi-Young Park, Dong Rak Kwon, Seok Won Jeong, Da Young Lee, Beom Soo Shin

**Affiliations:** 1 Center for Pharmacometrics and Systems Pharmacology, Department of Pharmaceutics, College of Pharmacy, University of Florida, Orlando, FL 32827, USA; taehwan.kim@cop.ufl.edu (T.H.K.); sarahkim@cop.ufl.edu (S.K.); JBulitta@cop.ufl.edu (J.B.B.); 2 College of Pharmacy, Wonkwang University, Iksan, Jeonbuk 54538, Korea; shins@wku.ac.kr; 3 College of Pharmacy, Catholic University of Daegu, 13-13 Hayang-ro, Hayang-eup, Gyeongsan-si, Gyeongbuk 38430, Korea; weonky@cu.ac.kr (K.-Y.W.); sjoo@cu.ac.kr (S.H.J.); masook@cu.ac.kr (E.M.); jswpia@gmail.com (S.W.J.); dayoung0717@gmail.com (D.Y.L.); 4 School of Pharmacy, Sungkyunkwan University, Suwon, Gyeonggi-do 16419, Korea; sdyoo@skku.ac.kr; 5 Department of Rehabilitation Medicine, School of Medicine, Catholic University of Daegu, Daegu 42472, Korea; parkgy@cu.ac.kr (G.-Y.P.); coolkwon@cu.ac.kr (D.R.K.)

**Keywords:** herbal medicine, drug-drug interaction, pharmacokinetics, gemcitabine, erlotinib, Hyangsayukgunja-tang, population pharmacokinetic modeling

## Abstract

Gemcitabine and erlotinib are the chemotherapeutic agents used in the treatment of various cancers and their combination is being accepted as a first-line treatment of advanced pancreatic cancer. Hyangsayukgunja-tang (HYT) is a traditional oriental medicine used in various digestive disorders and potentially helpful to treat gastrointestinal adverse effects related to chemotherapy. The present study was aimed to evaluate the effect of HYT on the pharmacokinetics of gemcitabine and erlotinib given simultaneously in rats. Rats were pretreated with HYT at an oral dose of 1200 mg/kg/day once daily for a single day or 14 consecutive days. Immediately after pretreatment with HYT, gemcitabine and erlotinib were administered by intravenous injection (10 mg/kg) and oral administration (20 mg/kg), respectively. The effects of HYT on pharmacokinetics of the two drugs were estimated by non-compartmental analysis and pharmacokinetic modeling. The pharmacokinetics of gemcitabine and erlotinib were not altered by single dose HYT pretreatment. However, the plasma levels of OSI-420 and OSI-413, active metabolites of erlotinib, were significantly decreased in the multiple dose HYT pretreatment group. The pharmacokinetic model estimated increased systemic clearances of OSI-420 and OSI-413 by multiple doses of HYT. These data suggest that HYT may affect the elimination of OSI-420 and OSI-413.

## 1. Introduction

Pancreatic cancer is one of the most fatal malignancies; it is rarely diagnosed at an early stage [1,2], and currently the fourth leading cause of cancer-related deaths in the United States with a low five-year relative survival rate of 8% [3]. Despite the trend that the overall cancer death rate dropped for most cancers over the past decade, death rates due to pancreatic cancer increased by 0.3% and 0.1% per year in men and women, respectively [3,4]. Based on expected changes in both incidence and death rates among expected demographic shifts, pancreatic cancer is projected to rank second among the causes of cancer death by 2030 [4]. 

Gemcitabine has been used as the first-line therapy for patients with metastatic pancreatic cancer since 1996 as showing a nine times greater survival rate than that of 5-fluorouracil within a year after treatment [5,6]. Most clinical trials of gemcitabine-based combination therapies with cytotoxic or biologic agents failed to find advantageous regimens [7,8,9]. In contrast to no improvement for other concomitant drugs with gemcitabine, the use of erlotinib in combination with gemcitabine has shown better efficacy than gemcitabine alone by increasing the one-year survival rate from 17% to 23% [8,10,11]. Erlotinib is a reversible tyrosine kinase inhibitor of the epidermal growth factor receptor (EGFR) and is commercially available as film-coated tablets [12]. After oral administration, erlotinib is mainly metabolized by liver CYP3A4 and CYP1A2 and found in the blood as O-demethylated isomers of either side chains OSI-413 and OSI-420 [13,14]. The in vitro inhibitory activities of these two metabolites against EGFR are comparable to that of erlotinib [14]. In 2005, the U.S. Food and Drug Administration (FDA) approved the combination therapy of erlotinib and gemcitabine for pancreatic cancer [15]. 

In addition to the chemotherapeutic agents, popularity and consumption of oriental herbal remedies have risen globally. A national survey reported approximately one in five adults in the United States takes herbal products for medical purposes [16,17,18]. Hyangsayukgunja-tang (HYT, named as “Xiang Sha Liu Jun Zi Tang” in Chinese) has been used in various digestive disorders, such as gastric flatulence, anorexia, nausea, and vomiting. HYT is commercially-available and consists of 14 herbs: Cyperi Rhizoma, Atractylodis Rhizoma Alba, Poria Sclerotium, Pinelliae Tuber, Citri Unshius Pericarpium, Amomi Fructus Rotundus, Magnoliae Cortex, Amomi Fructus, Ginseng Radix Alba, Aucklandiae Radix, Aipiniae Oxyphyllae Fructus, Glycyrrhizae Radix et Rhizoma, Zingiberis Rhizoma Crudus, and Zizyphi Fructus [19]. Marker compounds, such as liquiritin, hesperidin, ginsenoside Rg1, ginsenoside Rb1, glycyrrhizin, 6-gingerol, atractylenolide III, honokiol, costunolide, dehydrocostuslactone, atractylenolide II, nootkatone, and magnolol, were analyzed in HYT by LC-MS/MS [19]. In practice, chief chemical markers including hesperidin (1.4 mg/g), liquiritin (0.3 mg/g), gylcyrrhizin (0.06 mg/g), and 6-gingerol (0.4 mg/g) have been used for quality assessment [20]. 

Various pharmacological activities of HYT has been reported. In experimental animals, HYT showed protective effects on indomethacin-induced gastric mucosal lesions [21] and duodenal ulcer [22]. By regulating gut hormones, HYT also increased appetite in rats [23]. HYT significantly improved electrogastrogram, promoted gastrointestinal motility and gastric emptying, and decreased gastric sensitivity [24,25,26]. The meta-analysis of 15 randomized controlled trials found the superior efficacy of HYT for the treatment of functional dyspepsia compared to prokinetic drugs, such as domperidone, cisapride, and mosapride [25]. HYT was also effective in childhood abdominal pain [27]. Moreover, HYT has been reported to be effective in treatment pain, inflammation [24], and hyperlipidemia in animal models [28]. 

One of the most common adverse reactions associated with anticancer agents, including gemcitabine and erlotinib, are gastrointestinal disorders. The gastrointestinal disorders associated with erlotinib include diarrhea, anorexia, weight loss, stomatitis, oral ulceration, dyspnea, nausea, and vomiting [14]. Other adverse reactions include fatigue, rash, cough, alopecia, hirsutism, etc. [14]. For gemcitabine, nausea and vomiting are most common, while pain, fever, rash, dyspnea, constipation, diarrhea, hemorrhage, and infection are also reported [29]. Patients receiving gemcitabine and erlotinib experienced higher frequencies of rash, diarrhea, infection, and stomatitis, as well [8]. Other adverse events associated with combination of erlotinib and gemcitabine treatment were similar with previous experience with both agents. 

Therefore, HYT could be used to improve symptoms associated with chemotherapy due to its various effects. Nevertheless, potential interactions between anticancer agents and herbal remedies are not well known. Concurrent use of the herbal remedies with conventional western drugs may mimic, augment, or oppose the pharmacokinetics or pharmacodynamics, leading to either increase or decrease of their effects [30], which requires attention. 

The present study aimed to examine the pharmacokinetic interaction of gemcitabine and erlotinib for the treatment of pancreatic cancer with the oriental herbal medicine HYT. Alterations in pharmacokinetics of gemcitabine and erlotinib by HYT were determined in rats and quantitatively assessed by population pharmacokinetic modeling.

## 2. Results

### 2.1. Pharmacokinetics of Gemcitabine and Its Metabolite dFdU in Rats Pretreated with HYT

**Figure 1:**
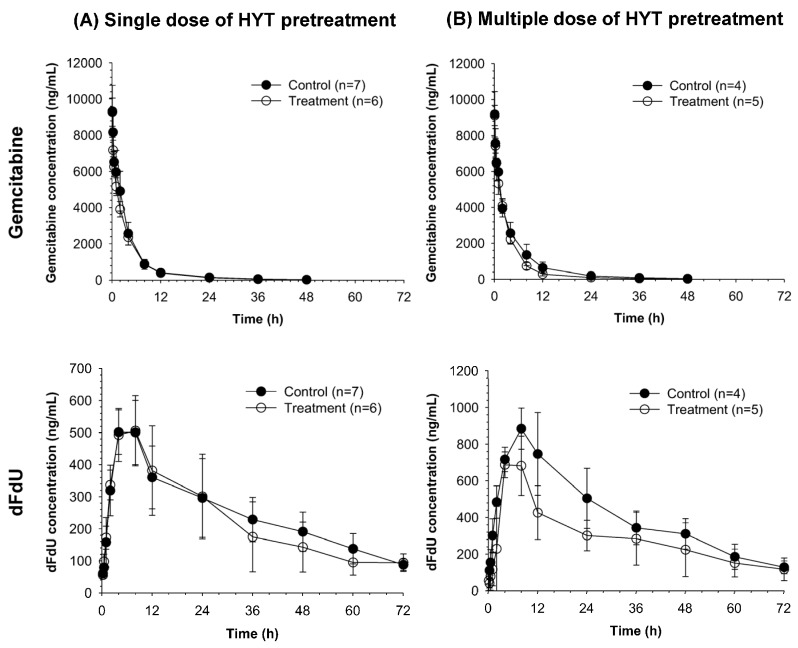
Average plasma concentration vs. time profiles of gemcitabine and dFdU following administration of the combination of anticancer agent; gemcitabine (i.v., 10 mg/kg) and erlotinib (p.o., 20 mg/kg) to rats pretreated with 1% CMC-Na (Control) or Hyangsayukgunja-tang (HYT) 1200 mg/kg as (**A**) a single dose or (**B**) multiple doses for 14 days.

The average plasma concentrations vs. time profiles of gemcitabine and its metabolite, 2′,2′-difluorodeoxyuridine (dFdU) after co-administration of gemcitabine (i.v., 10 mg/kg) and erlotinib (p.o., 20 mg/kg) in rats pretreated with HYT for one day or 14 days are shown in Figure 1. The average non-compartmental pharmacokinetic parameters of gemcitabine and dFdU are summarized in Table 1.

The overall plasma concentration vs. time profiles of gemcitabine and dFdU in the single-dose HYT pretreated group were comparable with those in the control group (Figure 1A). The slightly longer elimination half-life (t_1/2_) and greater volume of distribution (V_z_/F) of gemcitabine were observed in the single-dose HYT pretreated group. However, other pharmacokinetic parameters for both gemcitabine and dFdU were all similar between the single-dose HYT pretreated and control group (Table 1).

Similarly, the overall plasma concentration vs. time profiles of gemcitabine and dFdU were not significantly altered by multiple dose HYT pretreatment (Figure 1B). While T_max_ of dFdU was decreased, other non-compartmental pharmacokinetic parameters for gemcitabine and dFdU were comparable between the multiple-dose HYT-pretreated and control groups (Table 1).

### 2.2. Pharmacokinetics of Erlotinib and Its Metabolites OSI-420 and OSI-413 in Rats Pretreated with HYT 

**Figure 2:**
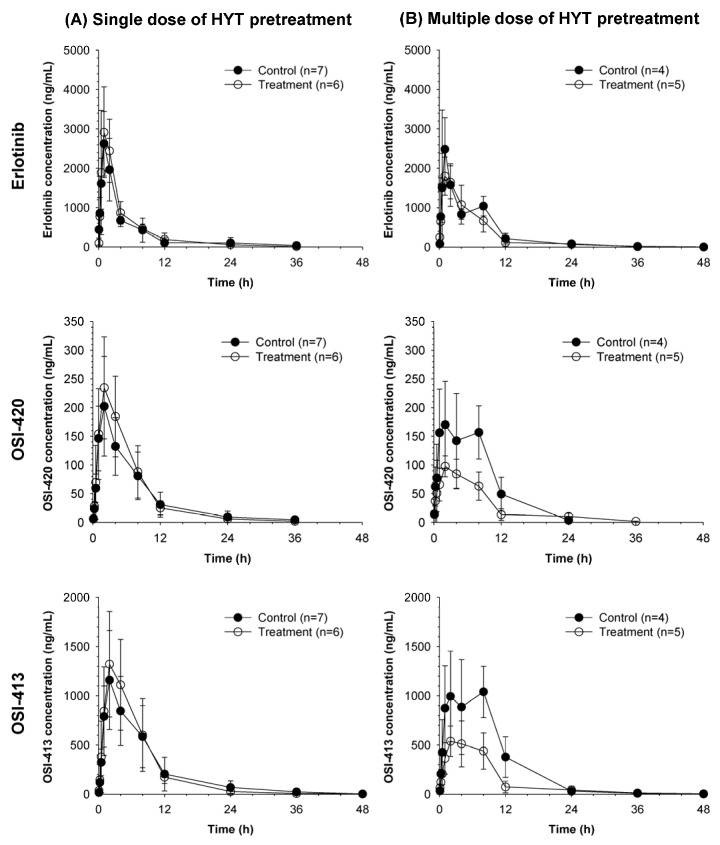
Average plasma concentration vs. time profiles of erlotinib, OSI-420, and OSI-413 following administration of the combination of anticancer agent; gemcitabine (i.v., 10 mg/kg) and erlotinib (p.o., 20 mg/kg) to rats pretreated with 1% CMC-Na (Control) or Hyangsayukgunja-tang (HYT) 1200 mg/kg as (**A**) a single dose or (**B**) multiple doses for 14 days.

The average plasma concentrations vs. time profiles of erlotinib and its metabolites, OSI-420 and OSI-413, after concurrent administration of gemcitabine (i.v., 10 mg/kg) and erlotinib (p.o., 20 mg/kg) in rats pretreated with HYT for one day or 14 days are shown in Figure 2. The average non-compartmental pharmacokinetic parameters of erlotinib, OSI-420, and OSI-413 are summarized in Table 2. 

The overall plasma concentration profiles of erlotinib, as well as its metabolism to OSI-420 and OSI-413, were not significantly altered by HYT single-dose pretreatment (Figure 2A) and pharmacokinetic parameters of erlotinib, OSI-420, and OSI-413 in single does HYT pretreated group were also comparable with those in control group (Table 2). 

In multiple dose HYT pretreated group, there were no significant differences in pharmacokinetic parameters of erlotinib compared to those in control rats. In contrast, OSI-420 and OSI-413 showed lower peak plasma concentrations (C_max_), as well as the area under the plasma concentration-time curve (AUC) values in the multiple-dose HYT-pretreatment group (Figure 2B, Table 2). The AUC ratio of each metabolite and erlotinib (AUC_meta_/AUC_parent_) decreased to 66.7% for OSI-420 and 57.3% for OSI-413 by multiple doses of HYT compared to those in control. On the other hand, there were no significant differences in elimination t_1/2_ of OSI-420 and OSI-413 between control and the HYT multiple-dose pretreated group.

### 2.3. Pharmacokinetic Modeling

Since gemcitabine and dFdU plasma levels did not change significantly by HYT administrations, a pharmacokinetic model was developed focusing on the pharmacokinetics of erlotinib and its active metabolites, OSI-420 and OSI-413. The reduced plasma levels of OSI-420 and OSI-413 may be due to the decreases in their formation or increases in their elimination. Either possibilities were tested by comparing the model performance by separately estimating the formation clearance of the metabolites, i.e., CL_erlotinib_·F_met_ (Model 1) or systemic clearances of the metabolites, i.e., CL_OSI420_ and CL_OSI413_ (Model 2) for control and 14-day HYT-treated groups. Other parameters were shared by the two groups. As a result, the model with separate systemic clearances of the metabolites (Model 2) was found to be superior to Model 1 in describing all the plasma concentration-time data from control and HYT pretreated groups and selected as the final model. 

**Figure 3:**
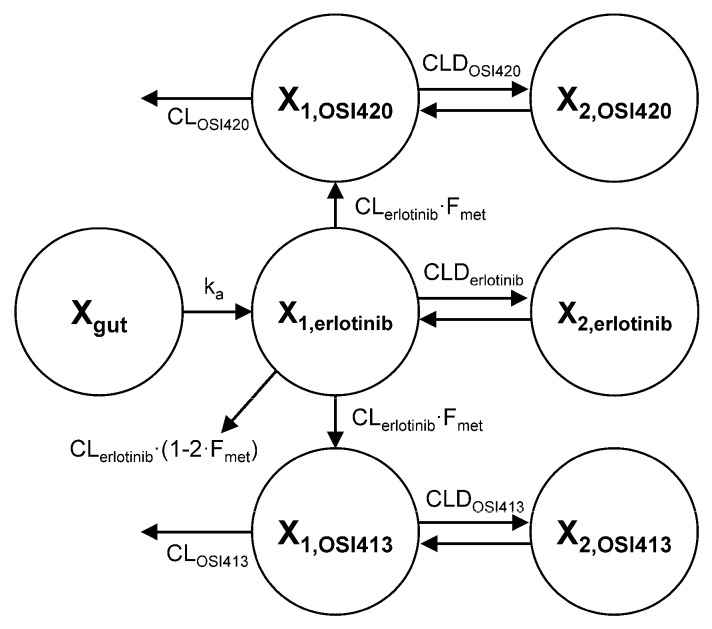
Structural model for the absorption and disposition of erlotinib and its active metabolites, OSI-420 and OSI-413 in rats.

**Figure 4:**
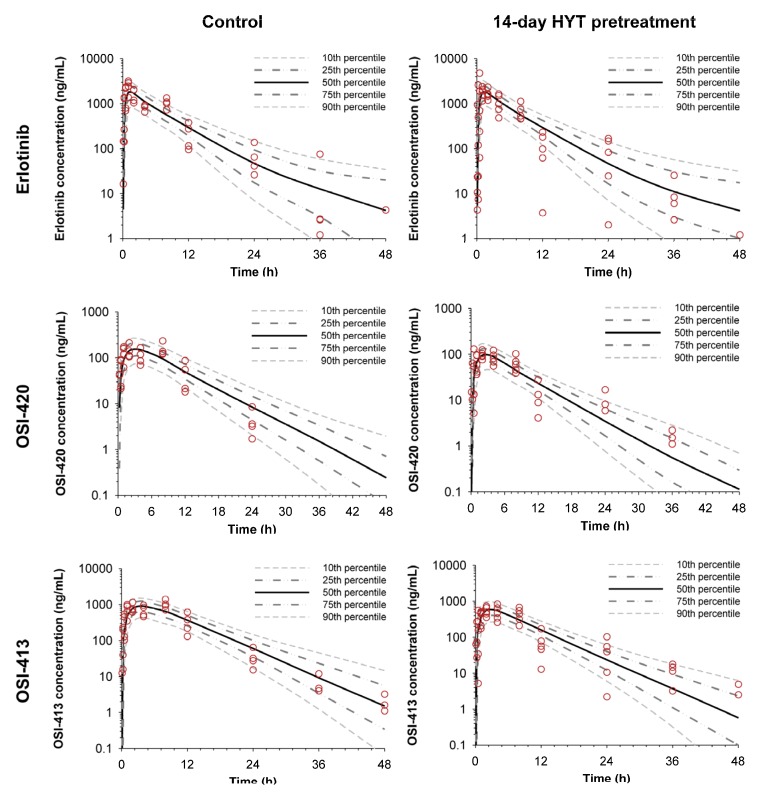
Visual predictive check plots of the population pharmacokinetic model for erlotinib (**upper**), OSI-420 (**middle**), and OSI-413 (**lower**). The observed plasma concentration of erlotinib, OSI-420, and OSI-413 in control and 14-day HYT pretreatment group (open circles) are shown with the lines representing the 10th, 25th, 50th, 75th, and 90th percentiles of the population predictions.

The final structural model for the absorption and disposition of erlotinib and the metabolites is shown in Figure 3. As indicated by the visual predictive check plots (Figure 4), the developed model showed reasonable predictability compared to the observed values. The population pharmacokinetic parameter estimates are summarized in Table 3. The estimated CL_OSI420_ and CL_OSI413_ of the 14-day HYT-pretreated group was 1.88-fold and 1.92-fold higher, respectively, than that of the control group (Table 3), which is consistent with the decreased C_max_ and AUC of the metabolites obtained by non-compartmental analysis.

## 3. Discussion

An increasing number of patients consider the use of complementary and alternative medicines in combination with their conventional treatment. Among cancer patients, 7–48% have reported taking herbal medicines expecting their anti-cancer effect, improvement of cancer-related symptoms, and relieving chemotherapy-related adverse effects [31]. There are also increasing efforts to treat diseases, control symptoms, and improve patient’s quality of life by a combination of western drugs with oriental herbal medicines in clinical practice. However, concurrent use of herbal medicines with pharmaceutical drugs may increase or decrease the pharmacological or toxicological effects. Better understanding of the pharmacokinetics of the conventional medicine in combination with herbal medicines would help in elucidating the clinical efficacy and predicting various events related to the efficacy and toxicity [32].

This study examined the potential interactions of the chemotherapeutic agent gemcitabine and erlotinib for the treatment of pancreatic cancer and a traditional herbal medication, HYT, in rats by means of both non-compartmental analysis and population pharmacokinetic modeling. The plasma concentrations vs. time profiles of gemcitabine and its metabolite dFdU and erlotinib and its metabolites OSI-420 and OSI-413 were determined in rats pretreated with HYT and the effects of HYT was quantitatively evaluated. 

HYT has been traditionally used in Asian countries for the treatment of various digestive disorders and spleen deficiency syndrome [25] and is commercially available. Various effects of HYT have also been reported, including analgesic, anti-inflammation, and antioxidant activities [23,24]. Thus, HYT may be helpful to improve adverse symptoms associated with chemotherapy. Indeed, a multicenter clinical study has shown that the quality of life, including pain and anorexia in patients with pancreatic cancer, were improved by co-administration of HYT [33].

Our data indicated that a single dose HYT did not significantly alter the pharmacokinetics of gemcitabine, erlotinib, and their metabolites. In addition, the pharmacokinetics of gemcitabine and dFdU were not significantly affected by HYT multiple administrations for 14 days. However, pretreatment with 14-day multiple doses of HYT resulted in significant decrease in C_max_ and AUC of OSI-420 and OSI-413 without substantial changes in their parent drug, erlotinib plasma concentrations. 

The decreased AUC and C_max_ of the two erlotinib metabolites, i.e., OSI-420 and OSI-413 in rats pretreated with HYT multiple doses may be either a result from their reduced formation or increased elimination. However, the formation rates of OSI-420 and OSI-413, i.e., elimination rate of erlotinib were not altered by HYT multiple doses represented by unchanged pharmacokinetics of erlotinib, including t_1/2_ compared with control. Thus, the decrease in AUC and C_max_ of OSI-420 and OSI-413 is likely due to their own increased elimination. The increased elimination is usually reflected by decreased elimination t_1/2_. Nevertheless, the t_1/2_ of OSI-420 and OSI-413 were unchanged. The unchanged t_1/2_ of OSI-420 and OSI-413 may be because they follow flip-flop kinetics, where their formation is slower than their elimination. The flip-flop phenomenon is common for metabolites that are more hydrophilic than their parent drug and easily excreted. In flip-flop kinetics, the terminal phase of the drugs is determined not by their elimination but by their formation. Thus, although the eliminations of OSI-420 and OSI-413 were enhanced, they may not be reflected to their apparent t_1/2_.

To further evaluate the HYT effect on pharmacokinetics of erlotinib and its metabolites, we developed a population pharmacokinetic model. Population pharmacokinetic modeling has been suggested to be a useful tool for evaluation of drug-drug or herb-drug interactions [30]. The present pharmacokinetic model (Figure 3) was designed to describe the absorption, disposition, and elimination of erlotinib, OSI-420, and OSI-413. The predicted concentration vs. time profiles of erlotinib and its two metabolites in the plasma showed good agreement with observed data (Figure 4). The population pharmacokinetic modeling analysis also supported that the decrease in C_max_ and AUC of OSI-420 and OSI-413 may be attributed to their enhanced elimination by multiple dosing of HYT. The model estimated the higher clearances of OSI-420 (1.88-fold) and OSI-413 (1.92-fold) for HYT-pretreated rats than those for control rats, which are comparable with the magnitude of the decrease in AUC_all_ of OSI-420 and OSI-413. It also demonstrated that the formation of the metabolites from erlotinib were not significantly affected by HYT. A model with different metabolic clearances of erlotinib in the control and HYT-pretreated groups could not describe the observed plasma concentration-time data.

After oral absorption, erlotinib is extensively metabolized by liver CYP3A4 and CYP1A2 [13,14]. The major active metabolites are OSI-413 and OSI-420, which are O-demethylated isomers of either side chains [34] and the metabolic pattern is similar across species [12]. The lower exposure and higher clearance of OSI-420 following erlotinib administration were observed in smokers compared with nonsmokers, which is probably due to the induction of the enzyme metabolizing erlotinib by cigarette smoking [14,35]. Although drug interactions of erlotinib with CYP3A4 inducers and inhibitors are known, which may require dose adjustments [14], little information is available on drug interactions regarding its active metabolites, OSI-420 and OSI-413.

The mechanism by which multiple doses of HYT induces lower plasma levels of OSI-420 and OSI-413 remain unclear. It is speculated that various constituents of HYT may act as enzyme inhibitors or inducers for OSI-420 and OSI-413 metabolism leading to the altered plasma levels. Although OSI-420 and OSI-413 are known to be further metabolized, their complete elimination pathways are currently unknown. Further studies regarding the metabolic pathways of OSI-420 and OSI-413 and the effects of HYT on the enzyme inhibition or induction may help to understand underlying mechanisms of the current findings.

Unlike the metabolites of erlotinib, the metabolite of gemcitabine, dFdU pharmacokinetics did not show flip-flop kinetics. The elimination t_1/2_ of dFdU is much longer than that of gemcitabine itself (24.4–41.5 vs. 7.9–12.1 h). Thus, the terminal phase of the dFdU plasma concentration-time profile is likely determined by its own elimination, not by the formation from gemcitabine, i.e., disposition-rate limited. However, our data indicated that pharmacokinetics of gemcitabine and its metabolite dFdU were not changed by the administration of HYT.

In conclusion, these results suggest that even though the gemcitabine and erlotinib plasma levels were largely unchanged, the traditional herbal prescription, HYT administrations, may increase the degradation of the active metabolites of erlotinib. Whether the decreased plasma levels of active metabolites by HYT would lead to significant changes in clinical efficacy with erlotinib and gemcitabine therapy needs to be further evaluated.

## 4. Materials and Methods

### 4.1. Materials

Gemcitabine hydrochloride and erlotinib hydrochloride were purchased from LC Laboratories (Woburn, MA, USA). 2′,2′-Difluoro-2′-deoxyuridine (dFdU) was purchased from Toronto Research Chemicals Inc. (North York, ON, Canada). Desmethyl erlotinib was purchased from Santa Cruz Biotechnology Inc. (Santa Cruz, CA, USA). Hyangsayukgunja-tang (HYT) was obtained from Hankook Shinyak Corp. (Nonsan, Chungnam, Korea). Carboxy methyl cellulose sodium (CMC-Na) and methyl cellulose (MC) were obtained from Junsei Chemical (Tokyo, Japan). Acetonitrile and distilled water (all HPLC grades) were purchased from J.T. Baker, Inc. (Phillipsburg, NJ, USA) and formic acid was obtained from Aldrich Chemical Co. (Milwaukee, WI, USA).

### 4.2. Animal Study

The animal study was approved by the Ethics Committee for the Treatment of Laboratory Animals at Catholic University of Daegu and conducted following the standard operating procedures (SOPs). Male Sprague–Dawley rats (eight weeks, 280–300 g; Samtako, Osan, Korea) were kept in plastic cages with free access to a standard diet (Samtako, Osan, Korea). The animals were maintained at a temperature of 23 ± 2 °C with a 12 h light-dark cycle and relative humidity of 50 ± 10%. 

To examine the HYT effect on the pharmacokinetics of gemcitabine and erlotinib, HYT was pretreated in rats as a single or once daily for 14 consecutive days. For pretreatment, dried, extracted HYT was suspended in 1% CMC-Na and given at a dose of 1200 mg/kg, while control rats received the HYT-free dosing vehicle. The clinical dose of HYT is 9 g/day which corresponds to 128.6 mg/kg/day for 70 kg human. Approximately a 10-fold higher HYT dose of its clinical dose was used in rats in the present study. HYT over 1000 mg/kg dose has been reported to show various pharmacological effects in animals [22,36]. 

Prior to gemcitabine and erlotinib administration, the rats were fasted overnight. Immediately after the HYT dose, 10 mg/kg of gemcitabine dissolved in saline was intravenously injected and 20 mg/kg of erlotinib suspended in 0.5% MC was orally administered. Following administration or gemcitabine and erlotinib, 0.3 mL of blood samples were collected at predose, 5, 15, 30 min, and 1, 2, 4, 8, 12, 24, 36, 48, 60, and 72 h post-dose from the jugular vein. Plasma samples were harvested by centrifugation of the blood samples at 4000× *g* for 10 min and stored at −80 °C until analysis.

### 4.3. LC-MS/MS

Gemcitabine and dFdU concentrations in rat plasma were simultaneously determined by a liquid chromatography-tandem mass spectrometry (LC-MS/MS). The LC-MS/MS comprised an API 4000 triple quadrupole mass spectrometer (AB MDS Sciex, Toronto, ON, Canada) coupled with an Agilent 1100 HPLC system (Agilent, Santa Clara, CA, USA). Plasma samples were separated on a Synergi Fusion-RP C_18_ Column (100 mm × 2.0 mm i.d., 2.5 μm) and SecurityGuard Guard Cartidge (Phenomenex, Torrence, CA, USA). An isocratic solvent system consisting of acetonitrile and 0.05% aqueous formic acid (20:80 *v*/*v* %) with 0.3 mL/min of flow rate was used as the mobile phase. The column oven temperature was 30 °C and the total run time was 4.5 min. The mass spectrometer was operated using electron spray ionization (ESI) in positive ion mode with mass transition at 264.2→112.2 for gemcitabine and 172.2→154.2 for gabapentin, the internal standard and negative ion mode with a mass transition at 262.8→220.2 for dFdU. The plasma samples were prepared by the protein precipitation method using methanol. Gabapentin working solution (100 ng/mL in methanol) 50 μL and 100 μL of methanol as a precipitation agent were added to 50 μL of the plasma samples. The mixture was vortexed for 1 min and centrifuged for 10 min at 4000× *g*. The supernatant (100 μL) was mixed with an identical volume of water and 10 μL of the final mixture was injected onto the LC-MS/MS. Analyst 1.4 software (AB MSD Sciex, Toronto, ON, Canada) was used for the data acquisition. The lower limit of quantification was 5 ng/mL for gemcitabine and 10 ng/mL for dFdU and the assay was validated using quality control (QC) samples.

Erlotinib and desmethyl erlotinib metabolites concentrations in rat plasma were simultaneously determined by LC-MS/MS. The LC-MS/MS comprised an Agilent 6430 mass spectrometer coupled with an Agilent 1200 HPLC system (Agilent, Santa Clara, CA, USA). Plasma samples were separated on a Kinetex C_18_ Column (50 mm × 2.1 mm i.d., 2.6 μm) and a SecurityGuard Guard Cartridge (Phenomenex, Torrence, CA, USA). An isocratic solvent system consisting of acetonitrile and 0.05% aqueous formic acid (30:70 *v*/*v* %) with a 0.3 mL/min flow rate was used as the mobile phase. The column oven temperature was 30 °C and the total run time was 3.5 min. The mass spectrometer was operated using electron spray ionization (ESI) in positive ion mode with a mass transition at 394.2→278.1 for erlotinib and at 544.2→397.1 for doxorubicin, the internal standard and negative ion mode with a mass transition at 380.2→278.1 for OSI-413 and OSI-420. The plasma samples were prepared by the protein precipitation method using methanol. Doxorubicin working solution (500 ng/mL in methanol) (50 μL) and 100 μL of methanol as a precipitation agent were added to 50 μL of the plasma samples. The mixture was vortexed for 1 min and centrifuged for 10 min at 4000× *g*. The supernatant (100 μL) was mixed with an identical volume of water and 1 μL of the final mixture was injected onto the LC-MS/MS. MassHunter Quantitative Analysis (Agilent Technologies, Santa Clara, CA, USA) was used for the data acquisition. The lower limits of quantification were 1 ng/mL for erlotinib and its metabolites and the assay was validated using QC samples.

### 4.4. Non-Compartmental Pharmacokinetic Analysis

The pharmacokinetic parameters were determined by non-compartmental analysis using Phoenix^®^ WinNonlin^®^ 6.4 (Certara, L.P., Princeton, NJ, USA). These parameters included the apparent clearance (CL/F), terminal half-life (t_1/2_), apparent volume of distribution (V_z_/F), and the area under the plasma concentration–time curve from time zero to the last observation time point (AUC_all_) and to infinity (AUC_inf_). The maximum plasma concentration (C_max_) and the time to reach C_max_ (T_max_) were obtained directly from observational data. Data was presented as the mean ± standard deviation (SD).

### 4.5. Pharmacokinetic Modeling

To evaluate the HYT effect on pharmacokinetics of erlotinib and its metabolites, we developed a population pharmacokinetic model. The model structure is depicted in Figure 3. The structural model was designed to capture the absorption, disposition and elimination of erlotinib, OSI-420 and OSI-413. The oral absorption of erlotinib was described as a first order rate constant, k_a_ with lag time. The differential equation for erlotinib amount in the absorption site (X_gut_) was:

(1) $$\frac{{\mathrm{dX}}_{\mathrm{gut}}}{\mathrm{dt}}=-k_{a}\cdot X_{\mathrm{gut}}$$

The systemic dispositions of erlotinib and its metabolites were tested using a linear model with a central and one or two peripheral compartments. It was decided that the final model use two systemic disposition compartments for respective parent drug and metabolites based on the objective function value. Erlotinib in the central compartment (X_1,erlotinib_) was distributed to the peripheral compartment (X_2,erlotinib_) and eliminated from central compartment. The differential equations for the amounts of erlotinib in these compartments were:

(2) $$\frac{{\mathrm{dX}}_{1,\text{erlotinib}}}{\mathrm{dt}}=k_{a}\cdot X_{\mathrm{gut}}-{\mathrm{CLD}}_{\text{erlotinib}}\cdot C_{1,\text{erlotinib}}+{\mathrm{CLD}}_{\text{erlotinib}}\cdot C_{2,\text{erlotinib}}-{\mathrm{CL}}_{\text{erlotinib}}\cdot C_{1,\text{erlotinib}}$$

(3) $$\frac{{\mathrm{dX}}_{2,\text{erlotinib}}}{\mathrm{dt}}={\mathrm{CLD}}_{\text{erlotinib}}\cdot C_{1,\text{erlotinib}}-{\mathrm{CLD}}_{\text{erlotinib}}\cdot C_{2,\text{erlotinib}}$$

where C_1,erlotinib_ and C_2,erlotinib_ represented erlotinib concentrations in their respective compartments and CL_erlotinib_ and CLD_erlotinib_ were systemic and distribution clearances of erlotinib, respectively. A fraction (F_met_) of the erlotinib in X_1,erlotinib_ was metabolized to OSI-420 (X_1,OSI420_) and OSI-413 (X_1,OSI413_), which was assumed to 0.1 for both metabolism. The metabolites were distributed to their respective peripheral compartments (X_2,OSI420_ and X_2,OSI413_) and eliminated from the central compartments. The differential equations for the amounts of OSI-420 and OSI-413 in the central and peripheral compartments were:

(4) $$\frac{{\mathrm{dX}}_{1,\mathrm{OSI}420}}{\mathrm{dt}}=F_{\mathrm{met}}\cdot {\mathrm{CL}}_{\mathrm{erlotinib}}-{\mathrm{CLD}}_{\mathrm{OSI}420}\cdot C_{1,\mathrm{OSI}420}+{\mathrm{CLD}}_{\mathrm{OSI}420}\cdot C_{2,\mathrm{OSI}420}-{\mathrm{CL}}_{\mathrm{OSI}420}\cdot C_{1,\mathrm{OSI}420}$$

(5) $$\frac{{\mathrm{dX}}_{2,\mathrm{OSI}420}}{\mathrm{dt}}={\mathrm{CLD}}_{\mathrm{OSI}420}\cdot C_{1,\mathrm{OSI}420}-{\mathrm{CLD}}_{\mathrm{OSI}420}\cdot C_{2,\mathrm{OSI}420}$$

C_1,OSI420_ and C_2,OSI420_ represented OSI-420 concentrations in their respective compartments and CL_OSI420_ and CLD_OSI420_ were systemic and distribution clearances of OSI-420, respectively.

(6) $$\frac{{\mathrm{dX}}_{1,\mathrm{OSI}413}}{\mathrm{dt}}=F_{\mathrm{met}}\cdot {\mathrm{CL}}_{\text{erlotinib}}-{\mathrm{CLD}}_{\mathrm{OSI}413}\cdot C_{1,\mathrm{OSI}413}+{\mathrm{CLD}}_{\mathrm{OSI}413}\cdot C_{2,\mathrm{OSI}413}-{\mathrm{CL}}_{\mathrm{OSI}413}\cdot C_{1,\mathrm{OSI}413}$$

(7) $$\frac{{\mathrm{dX}}_{2,\mathrm{OSI}413}}{\mathrm{dt}}={\mathrm{CLD}}_{\mathrm{OSI}413}\cdot C_{1,\mathrm{OSI}413}-{\mathrm{CLD}}_{\mathrm{OSI}413}\cdot C_{2,\mathrm{OSI}413}$$

C_1,OSI413_ and C_2,OSI413_ represented OSI-413 concentrations in their respective compartments and CL_OSI413_ and CLD_OSI413_ were systemic and distribution clearances of OSI-413, respectively.

The plasma concentrations of erlotinib, OSI-420 and OSI-413 were fitted simultaneously by the population pharmacokinetic modeling using the importance sampling version of the Monte Carlo Parametric Expectation Maximization (MC-PEM) algorithm in the parallelized S-ADAPT software (version 1.57). An importance sampling MC-PEM method (pmethod = 4 in S-ADAPT) was used for the population parameter estimation and log-normal distribution was used to describe the between subject variability (BSV) for each parameter estimate. The goodness of fit was assessed by visual inspection of the observed and fitted concentrations, the objective function, plausibility of parameter estimates, standard diagnostic plots, the normalized prediction distribution error, and visual predictive checks. 

### 4.6. Statistical Analysis

Statistical analysis was conducted using SPSS software (Version 17.0, IBM Co., NY, USA). The obtained parameters were compared by unpaired *t*-tests between the two means for unpaired data. *p* values < 0.05 were considered as statistically significant.

## Figures and Tables

**Table 1:** Non-compartmental pharmacokinetic parameters of gemcitabine and dFdU following administration of the combination of anticancer agent; gemcitabine (i.v., 10 mg/kg) and erlotinib (p.o., 20 mg/kg) in rats pretreated with 1% CMC-Na (Control) or Hyangsayukgunja-tang (HYT) 1200 mg/kg as a single dose or multiple doses for 14 days.

	Parameter	Single Dose		Multiple Doses	
		Control (*n* = 7)	HYT (*n* = 6)	Control (*n* = 4)	HYT (*n* = 5)
Gemcitabine	t_1/2_ (h)	7.9 ± 1.2	9.8 ± 0.8 *	12.1 ± 2.1	10.6 ± 2.4
	C_0_ (ng/mL)	10,067.5 ± 2101.4	10,562.9 ± 1241.2	10,163.2 ± 1938.0	10,098.7 ± 820.5
	AUC_all_ (ng·h/mL)	34,684.2 ± 6088.4	30,934.6 ± 4475.8	37,842.2 ± 9170.3	29,383.8 ± 3473.1
	AUC_inf_ (ng·h/mL)	34,931.4 ± 6017.6	31,222.8 ± 4518.1	38,585.6 ± 9292.4	29,705.9 ± 3561.8
	V_z_/F (L/kg)	3.4 ± 0.9	4.6 ± 1.0 *	4.9 ± 2.0	5.2 ± 1.4
	CL/F (mL/min/kg)	4.9 ± 1.0	5.4 ± 0.8	4.5 ± 1.1	5.7 ± 0.7
	V_ss_ (L/kg)	1.7 ± 0.3	1.9 ± 0.1	2.1 ± 0.3	1.9 ± 0.1
dFdU	t_1/2_ (h)	29.2 ± 7.2	41.5 ± 28.6	24.4 ± 3.9	29.6 ± 5.7
	T_max_ (h)	6.3 ± 3.1	7.3 ± 3.0	9.0 ± 2.0	5.6 ± 2.2 *
	C_max_ (ng/mL)	550.7 ± 68.1	538.3 ± 93.9	885 ± 112.5	722 ± 141.8
	AUC_all_ (ng·h/mL)	16,597.2 ± 2484	16,228.1 ± 5943.2	27,104.5 ± 2154.8	20,919.8 ± 7518.3
	AUC_inf_ (ng·h/mL)	22,137.7 ± 5022.7	21,221.6 ± 6950.8	35,453.2 ± 7118.4	25,824.8 ± 9709
	AUC_meta_/AUC_parent_	0.64 ± 0.14	0.67 ± 0.18	0.74 ± 0.14	0.70 ± 0.17

* *p* < 0.05 vs. Control.

**Table 2:** Non-compartmental pharmacokinetic parameters of erlotinib, OSI-420, and OSI-413 following administration of the combination of anticancer agent; gemcitabine (i.v., 10 mg/kg) and erlotinib (p.o., 20 mg/kg) to rats pretreated with 1% CMC-Na (Control) or Hyangsayukgunja-tang (HYT) 1200 mg/kg as a single dose or multiple doses for 14 days.

	Parameter	Single Dose		Multiple Doses	
		Control (*n* = 7)	HYT (*n* = 6)	Control (*n* = 4)	HYT (*n* = 5)
Erlotinib	t_1/2_ (h)	5.9 ± 4.0	5.3 ± 2.6	4.3 ± 1.8	6.8 ± 3.5
	T_max_ (h)	0.8 ± 0.4	1.1 ± 0.5	1.0 ± 0.0	1.5 ± 0.7
	C_max_ (ng/mL)	2653.8 ± 760.3	3092 ± 1142.4	2484.5 ± 797.4	2449.3 ± 1390
	AUC_all_ (ng·h/mL)	11,544.5 ± 2210.6	13,419.5 ± 2713.9	14,218.5 ± 3731.5	12,422.6 ± 1026.3
	AUC_inf_ (ng·h/mL)	11,811.6 ± 2211.8	13,517 ± 2678.2	14,237.8 ± 3737.1	12,509.4 ± 1022.4
	CL/F (mL/min/kg)	14.5 ± 9.8	12.1 ± 7.1	9.1 ± 3.2	15.7 ± 7.4
	V_z_/F (L/kg)	29.1 ± 5.5	25.7 ± 6.5	24.7 ± 6.6	26.8 ± 2.4
OSI-420	t_1/2_ (h)	6.8 ± 4.5	3.9 ± 2.2	3.6 ± 0.8	5.9 ± 4.0
	T_max_ (h)	2.0 ± 0.0	2.3 ± 0.8	4.8 ± 3.8	3.7 ± 2.8
	C_max_ (ng/mL)	202.3 ± 87.1	238.1 ± 88.7	167.3 ± 44.7	114.7 ± 15.6 *
	AUC_all_ (ng·h/mL)	1553.3 ± 386.7	1568.5 ± 393.2	1666.5 ± 663.4	865 ± 223.8 *
	AUC_inf_ (ng·h/mL)	1598.6 ± 368.3	1617.4 ± 410.2	1689.9 ± 662	955.6 ± 171.5
	AUC_meta_/AUC_parent_	0.14 ± 0.03	0.12 ± 0.02	0.12 ± 0.03	0.08 ± 0.01 *
OSI-413	t_1/2_ (hr)	7.1 ± 5.8	5.8 ± 2.7	5.6 ± 1.4	5.7 ± 0.9
	T_max_ (hr)	2.9 ± 2.3	3.3 ± 2.4	4.8 ± 3.8	3.7 ± 2.8
	C_max_ (ng/mL)	1164.6 ± 493.7	1349.3 ± 508.9	1039 ± 259.7	649.4 ± 115.6 *
	AUC_all_ (ng·h/mL)	10,044.5 ± 3162.8	10,300.6 ± 3230.6	11,199.6 ± 3637.9	5671.4 ± 1018.4 *
	AUC_inf_ (ng·h/mL)	10,196.9 ± 2995.2	10,334.4 ± 3228.5	11,219.4 ± 3647.3	5697.5 ± 1007.6 *
	AUC_meta_/AUC_parent_	0.88 ± 0.28	0.83 ± 0.27	0.82 ± 0.17	0.47 ± 0.07 *

* *p* < 0.05 vs. Control.

**Table 3:** Population pharmacokinetic parameter estimates of erlotinib and its active metabolites, OSI-420 and OSI-413.

Parameter	Symbol	Unit	Population Mean (BSV)
Absorption rate constant for erlotinib	k_a_	1/h	1.14 (0.81)
Absorption lag time for erlotinib	T_lag_	h	0.155 (0.777)
Clearance for erlotinib	CL_erlotinib_/F	L/h/kg	1.5 (0.148)
Fraction of erlotinib clearance for metabolite formation	F_met_	-	0.1
Clearance for OSI-420 in control group	CL_OSI420,con_/F	L/h/kg	0.182 (0.174)
Clearance for OSI-420 in HYT pretreatment group	CL_OSI420,HYT_/F	L/h/kg	0.35 (0.122)
Clearance for OSI-413 in control group	CL_OSI413,con_/F	L/h/kg	1.18 (0.257)
Clearance for OSI-413 in HYT pretreatment group	CL_OSI413,HYT_/F	L/h/kg	2.22 (0.163)
Distribution clearance for erlotinib	CLD_erlotinib_/F	L/h/kg	2.68 (0.655)
Distribution clearance for OSI-420	CLD_OSI420_/F	L/h/kg	4.51 (0.0598)
Distribution clearance for OSI-413	CLD_OSI413_/F	L/h/kg	0.943 (0.0244)
Central volume of distribution for erlotinib	V_1,erlotinib_/F	L/kg	3.97 (0.0473)
Peripheral volume of distribution for erlotinib	V_2,erlotinib_/F	L/kg	3.91 (0.554)
Central volume of distribution for OSI-420	V_1,OSI420_/F	L/kg	0.0278 (0.316)
Peripheral volume of distribution for OSI-420	V_2,OSI420_/F	L/kg	1.88 (0.047)
Central volume of distribution for OSI-413	V_1,OSI413_/F	L/kg	0.00235 (0.359)
Peripheral volume of distribution for OSI-413	V_2,OSI413_/F	L/kg	0.4 (0.0394)
